# *Oncomelania hupensis* retains its ability to transmit *Schistosoma japonicum* 13 years after migration from permissive to non-permissive areas

**DOI:** 10.1186/s13071-020-4004-8

**Published:** 2020-03-19

**Authors:** Cheng-song Sun, Fang Luo, Xin Liu, Feng Miao, Wei Hu

**Affiliations:** 1 Department of Infectious Diseases, Huashan Hospital, State Key Laboratory of Genetic Engineering, Ministry of Education Key Laboratory for Biodiversity Science and Ecological Engineering, Ministry of Education Key Laboratory of Contemporary Anthropology, School of Life Sciences, Fudan University, Shanghai, 200438 People’s Republic Of China; 2 Anhui Provincial Institute of Parasitic Diseases, Hefei, 230061 Anhui Province People’s Republic Of China; 3 Shandong Institute of Parasitic Diseases, Shandong First Medical University & Shandong Academy of Medical Sciences, Jining, 272033 Shandong Province People’s Republic Of China; 4 National Institute of Parasitic Diseases, Chinese Center for Disease Control and Prevention, Key Laboratory of Parasite and Vector Biology of China Ministry of Health, WHO Collaborating Centre for Tropical Diseases, Joint Research Laboratory of Genetics and Ecology on Parasite-host Interaction, Chinese Center for Disease Control and Prevention & Fudan University, Shanghai, China

**Keywords:** *Schistosoma japonicum*, *Oncomelania hupensis*, Pathogenicity, Transcriptome, Differentially expressed genes, South-to-North Water Diversion Project

## Abstract

**Background:**

The East Route Project (ERP) of the South-to-North Water Diversion Project (SNWDP) stretches across schistosomiasis endemic and non-endemic areas in China, which may lead to the dispersal of *Oncomelania hupensis*, the intermediate host of *Schistosoma japonicum*, from permissive areas along the Yangtze River Basin to non-permissive areas in northern China. A previous survey demonstrated that *O. hupensis* could survive and breed for 13 years (12 generations) after being transferred to a non-permissive area, and could be infected by *S. japonicum*. However, it is not clear if the migrated snails will change their ability to transmit *S. japonicum*.

**Methods:**

We infected mice with the cercariae released from the infected transferred snails bred in Jining city of Shandong Province (non-permissive areas) for 13 years. The mice in the control group were infected with cercariae derived from the snails collected in their original habitat (Jiangdu county of Jiangsu Province, permissive areas). Then, we explored the pathogenicity to mice including worm burden, liver egg count and pathology. Additionally, the gene expression profiles of the adult male and female worms recovered from the infected mice were analyzed by RNA sequencing.

**Results:**

The worm burden, liver egg count and pathology of the mice infected with cercariae released from transferred snails bred in non-permissive areas for 13 years showed no significant differences, when compared with the control cercariae. Slight changes occurred at the transcription level between adult male and female worms recovered from mice infected with cercariae derived from snails bred in permissive and non-permissive areas. Only fourteen genes were significantly differentially expressed in the comparison of adult female worms, and no significantly differentially expressed gene was found in the comparison of adult male worms.

**Conclusions:**

Our findings strongly suggest that transferred snails did not change their schistosomiasis transmission ability and the worms derived from them retained the original pathogenicity, even after migrating from permissive to non-permissive areas for 13 years. Therefore, a long-term surveillance system of snails along the SNWDP is urgently needed to prevent the diffusion of *O. hupensis* and reduce the risk of transmission of schistosomiasis.
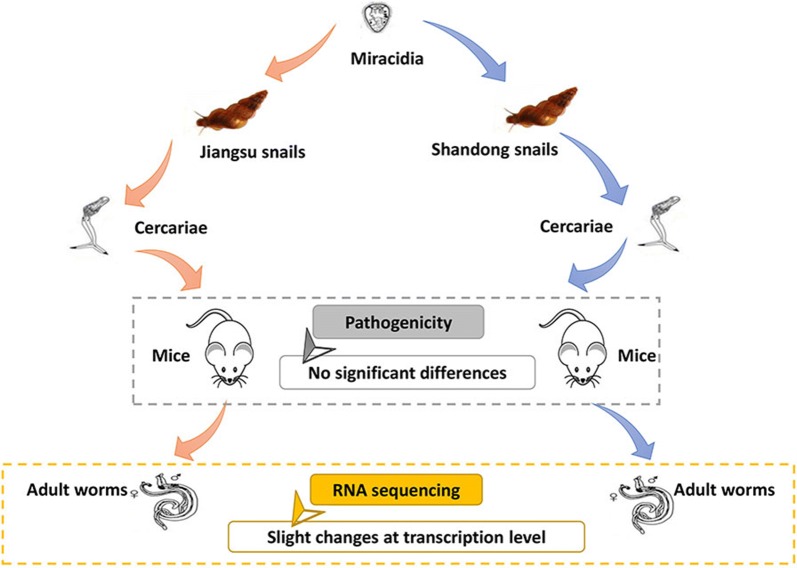

## Background

Schistosomiasis japonica, caused by trematode parasite *Schistosoma japonicum*, is a serious public health issue that leads to human morbidity and mortality, primarily in southern China and large parts of the Philippines [1, 2]. Over the past six decades, great achievements have been attained in reducing *S. japonicum* infections through comprehensive control measures [3, 4], but there were still 37,601 schistosomiasis patients in China at the end of 2017, including 1 acute and 29,407 advanced documented cases of schistosomiasis [5]. Over 90% of current schistosomiasis cases occur in the lake and marshland regions of Jiangsu, Jiangxi, Hunan, Hubei and Anhui provinces with vast areas of *Oncomelania hupensis* habitats [5], which greatly challenge the progress of schistosomiasis transmission control.

The amphibious freshwater snail *O. hupensis* is the only intermediate host of *S. japonicum* [6]. The snails become infected when the miracidia hatched from mature eggs penetrate into their bodies and then several larval stages develop, including sporocysts and cercariae [7]. The geographical distribution of snails defines the areas where schistosomiasis is endemic in China, so one of the main approaches is to interrupt the transmission of the disease to control the snails [8–10]. In China, *O. hupensis* is dominated at latitudes below 33° 15′ N [7]. Besides the environmental factors such as temperature, soil type and vegetation [11, 12], inevitably some water-conservancy projects affect the dispersal of snails and schistosomiasis epidemic, such as Aswan Reservoir in Egypt, Akosombo Dam in Ghana and Gezira Dam in Sudan [13–16].

The South-to-North Water Diversion Project (SNWDP), which was put into operation at the end of 2013, is a major strategic project to transfer part of the abundant water resources in the Yangtze River Basin to North and Northwest China. This action changes the situation regarding water shortages in North China, as well as providing flood prevention and drought eluviation in South China [17]. One of the main intakes of the Eastern Route Project (ERP) is in Jiangdu county of Jiangsu Province which is heavily infested with *O. hupensis* [18]. The route of ERP crosses Baoying county of Jiangsu Province (at 33° 15′ N), the current northern limit zone of *O. hupensis* distribution in China [19], and then passes northward into Shandong and Hebei provinces [17], non-permissive areas for *O. hupensis* survival. Following the construction of the project, studies have been carried out on whether *O. hupensis* will be brought to the north of China along with the water flow and form new snail habitats across 33° 15′ N, which then can eventually lead to the expansion of *S. japonicum* endemicity [20–25]. However, there is still a lack of long-term longitudinal observations to indicate whether *O. hupensis* can survive and reproduce in cold areas of northern China and maintain their ability to transmit schistosomiasis.

In our previous study, we transferred *O. hupensis* from Jiangdu county of Jiangsu Province (permissive areas) to Jining city of Shandong Province (non-permissive areas north of 33° 15′ N), where the ERP of SNWDP passes through. Unlike the results reported from other studies [20, 26–28], the snails survived and spawned for 13 years (12 generations) post-migration. During this time period we observed snails infected with miracidia and fed into the natural environment of the lakeshore. Interestingly, the offspring of the transferred snails continued to maintain the susceptibility to *S. japonicum* infection. In the present study, the cercariae released from the offspring snails were collected and used to infect mice. The worm burden, liver egg count and pathology showed no statistical differences at 50 days post-infection (dpi) compared with the cercariae derived from *O. hupensis* collected in Jiangdu county of Jiangsu Province which were infected with the same batch of miracidia of *S. japonicum* and bred under the same conditions. Furthermore, we utilized RNA sequencing to investigate the differences at the transcription level between adult *S. japonicum* derived from *O. hupensis* transferred to non-permissive areas and adult parasites derived from snails from their original permissive areas. Unsurprisingly, there were just slight changes at the transcription level. Our findings indicate that snails transferred from a permissive to a non-permissive area retain the original schistosomiasis transmission capacity. Therefore, a long-term surveillance system of a possible schistosomiasis epidemic along the ERP of SNWDP should be constructed to prevent the spread of snails and reduce the risk of schistosomiasis transmission.

## Methods

### Parasite and animals

**Fig. 1:**
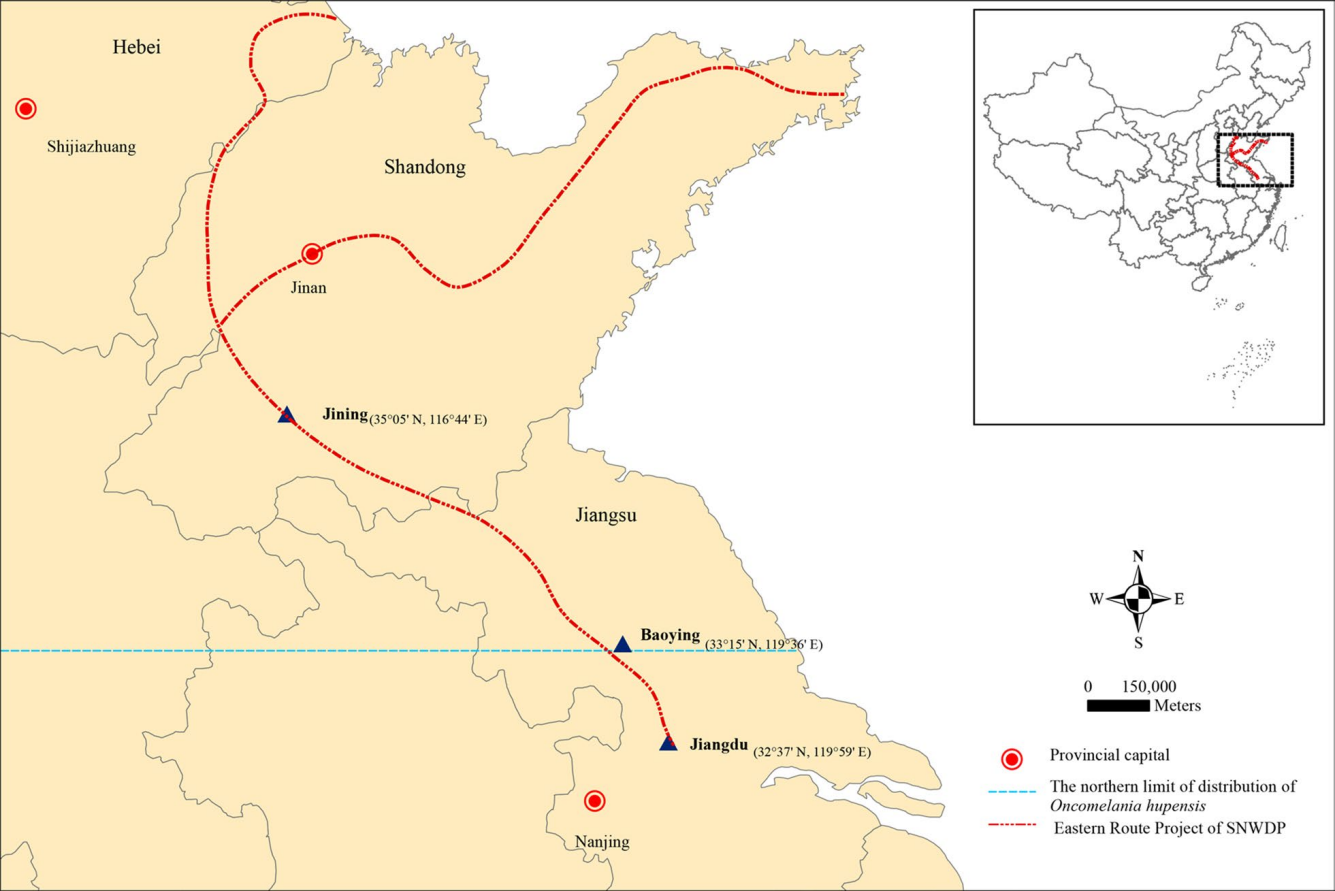
The location of breeding sites of *O. hupensis* in permissive (Jiangdu county of Jiangsu Province) and non-permissive areas (Jining city of Shandong Province) in China

*Oncomelania hupensis* were originally collected from Jiangdu county of Jiangsu Province (32° 37′ N, 119° 59′ E; hereinafter referred to as “Jiangsu snails”) near the ERP intake of SNWDP, and then transferred to a pond at the southern foot of Dushan Island, Lake Weishan, Jining City, Shandong Province (35° 05′ N, 116° 44′ E) (Fig. 1). The transferred snails were fed in the natural environment of the lakeshore, and survived and spawned for 13 years (12 generations, 2004–2017) (hereinafter referred to as “Shandong snails”). In the marshland of Lake Weishan, we rebuilt a natural environment isolated from adjacent areas as the experimental field, where the Jiangsu snails and Shandong snails were infected with miracidia of *S. japonicum* (Jiangsu isolate) and bred from July 3rd to November 10th 2017 (130 days). Infected Jiangsu snails and Shandong snails were selected to release cercariae and infect 12 and 13 ICR mice (age, 6–8 weeks; Shanghai SLAC laboratory Animal Co., Ltd., Shanghai, China; 20 cercariae per mouse), respectively. Mice were housed under specific pathogen-free conditions and fed autoclaved food and water *ad libitum*. Mice were maintained under environmentally controlled conditions (12:12h light:dark photocycle, temperature of 25 °C and relative humidity of 50%). At 50 dpi, mice were sacrificed, and the mature adult worms were recovered by hepatic perfusion. Male and female worms were separated manually under a dissecting microscope, counted for calculating worm burden and then pooled together into four groups: adult male and female worms derived from infected Jiangsu snails and Shandong snails (hereinafter referred to as “JS-M”, “JS-F”, “SD-M” and “SD-F”). There were three biological replicates in each group and twelve samples of worms were carefully washed and immediately frozen in liquid nitrogen until RNA extraction.

### Liver egg count and pathology

Livers of 4 and 6 mice infected by the cercariae were released from Jiangsu and Shandong snails, respectively. They were selected randomly for liver egg count and pathology analysis at 50 dpi. For each mouse, the right quadrate lobe of each liver was removed, weighed, homogenized and digested with 10% NaOH for 10 min at 56 °C. The eggs were counted and the number of eggs per gram of liver tissue per pair worm were calculated. To compare the liver pathology, the left quadrate lobe of each liver from the two groups was fixed in 4% formalin. Paraffin-embedded sections of these samples were prepared and stained with haematoxylin and eosin (H&E).

### RNA preparation

Total RNA was extracted from twelve samples of worms using TRIzol reagent (Invitrogen, CA, USA) and DNA contamination was removed by DNase (TaKaRa, Dalian, China) according to the manufacturer’s instructions. The total RNA concentration of each sample was measured using NanoDrop 2000c UV-Vis Spectrophotometer (Thermo Fisher Scientific Inc., Wilmington, USA). RNA integrity was confirmed by agarose gel electrophoresis and Agilent 2100 (Agilent Technologies, Santa Clara, California). Following the recommendation of the TruSeq™ RNA (Illumina, San Diego, USA) sample preparation guide, high-quality RNA samples (concentration > 50 ng/μl, RIN > 6.5, *28S*:*18S* ≥ 0.5, OD260/280 = 1.8–2.2, OD260/230 = 1.8–2.2) were used to construct cDNA libraries.

### cDNA library construction and RNA sequencing

Equal amounts of the twelve samples were prepared for cDNA synthesis and RNA sequencing. Before the construction of the cDNA libraries, ribosomal RNAs were removed and poly(A) + mRNAs were isolated with magnetic Oligo-dT beads (Invitrogen), then the libraries were constructed and sequenced by Majorbio Biotech Co., Ltd. (Shanghai, China). Briefly, the libraries were constructed by TruSeq^TM^ RNA Sample Prep Kit (Illumina) using 5 μg of the total RNA for each sample. The synthesized DNA templates were enriched by PCR amplification for 15 cycles. The products were collected and purified *via* Certified Low Range Ultra Agarose (Bio-Rad, Hercules, USA) gel electrophoresis. Before sequencing, the cDNA libraries were quantified using a TBS-380 micro fluorometer with Picogreen® reagent (Invitrogen). Clone clusters were produced on an Illumina cBot, using the TruSeq PE Cluster Kit v3-cBotHS, and high-throughput sequencing was carried out on the Illumina HiSeq4000 TruSeq SBS Kit v3-HS (200 cycles).

### Bioinformatics analysis of sequencing data

Clean reads were obtained with Trimmomatic v0.38 [29] by removing adapter sequences, empty reads, poly-N containing reads and low-quality sequences with a Q quality score lower than 20 from the raw data. Meanwhile, Q20, Q30 and GC-content of the clean reads were calculated. All of the downstream analysis was based on clean reads with high quality. The improved genome of *S. japonicum* [30] was used as the reference genome. An index of the reference genome was constructed and paired-end clean reads were aligned to the *S. japonicum* reference genome using HISAT v2.1.0 [31]. HTSeq v0.11.2 [32] was adopted to count the number of reads mapped to each gene. The genes with mapped reads of all samples > 10 were included for differential expression analysis. Differential expression analysis of the two groups (three replicates of each group) was performed using the *DESeq2* R package (1.18.0) [33]. The *P*-values were adjusted using the Benjamini and Hochberg’s approach for controlling the false discovery rate (FDR). Transcripts were determined to be significantly differentially expressed if FDR < 0.05 and > 2-fold change was observed in the level of expression.

Pearson’s correlation analysis used for RNA sequencing was performed to examine the correlation of gene expression levels between samples. The correlation of gene expression levels between samples is an important index to test the reliability of experiments and the rationality of sample selection. The closer the correlation coefficient is to 1, the higher the similarity of expression patterns between samples.

### qRT-PCR verification of RNA sequencing data

To validate the transcription levels of the genes identified by RNA sequencing, 14 genes (significantly differentially expressed between JS-F and SD-F) were measured using qRT-PCR, based on their involvement in different expression levels in all comparative analyses (JS-M *vs* SD-M, JS-F *vs* SD-F). The total RNA was treated with the PrimeScript™ RT reagent kit with gDNA Eraser (Takara, Dalian, China) to remove genomic DNA and synthesize cDNA template, according to the manufacturer’s instructions. Using the RNA sequencing data, primers used for qRT-PCR were designed using Primer Premier 6.0 software (Premier Biosoft International, Palo Alto, CA, USA); these are listed in Table 1. PSMD4 (26S proteasome non-ATPase regulatory subunit 4) was used as an internal reference gene [34]. All qPCR reactions were performed on a LightCycler® 96 (Roche, Basel, Switzerland) using 2× SYBR green qPCR master mix (Bimake, Houston, USA) according to the manufacturer’s instructions. Each 10 μl qPCR reaction mixture comprised a 1 μl (1:4) of cDNA, 5 μl 2× SYBR green master, 0.4 μl (5 μM) of each primer and 3.2 μl ddH_2_O. The qPCR cycle parameters were as follows: 95 °C for 5 min, followed by 40 cycles of 95 °C for 15 s, 60 °C for 30 s; melt curve analysis ranged from 60 °C to 95 °C to ensure that the specific product was amplified in each reaction. The 2^−ΔΔCq^ method was used to calculate the relative fold change of the differentially expressed transcripts [35]. All RNA samples for qRT-PCR validation were the same as those for the Illumina library synthesis.

**Table 1:** Primers used in the quantitative RT-PCR in the present study

Gene	Sequence (5′–3′)	Primer length (mer)	Tm (°C)	GC %	Product length (bp)
PSMD4	CCTCACCAACAATTTCCACATCT	23	55.50	43.50	129
	GATCACTTATAGCCTTGCGAACAT	24	55.80	41.70	
EWB00_005036	ATACAGAAGCAAGTCCTGAA	20	51.30	40.00	149
	CTCGCCTAATCCATCTTGT	19	53.01	47.37	
EWB00_010161	CTGTATCATAGTCGTCGTATC	21	53.66	42.86	104
	GCATCGTTCCTGTTCCTA	18	52.62	50.00	
EWB00_002418	TTATACGGTGGAAGCGAAT	19	50.85	42.11	114
	GGTGGGATATTAGGAGTTCAT	21	53.66	42.86	
EWB00_003584	TGCTATCGGACTCGTGTA	18	49.80	50.00	122
	GTAGAGACCATGTATGACAGT	21	50.10	42.90	
EWB00_003582	GCATTGAATACAGCAGGAC	19	49.70	47.40	136
	TTATAGCAACGGTTCGGTAT	20	50.00	40.00	
EWB00_008342	CAATCATCCACTTCAAGATACG	22	53.95	40.91	147
	CCAGCGAAGCAGGAGTAT	18	54.90	55.56	
EWB00_005617	GTGGTTCGCTACTGTCAT	18	52.62	50.00	145
	GATACTCACCGCAACTACA	19	53.01	47.37	
EWB00_005592	CTTATGCGTCGTGGATGA	18	52.62	50.00	102
	CCAGGATGACCAGAATGAA	19	53.01	47.37	
EWB00_010449	TCAATGTTGTCCTGAATGTG	20	51.30	40.00	100
	TTGTCTTGGTGTTCTTGGT	19	50.85	42.11	
EWB00_011245	CTGTCACACTACAACAAGAAG	21	53.66	42.86	126
	GTATTTCGTCACTGCCTTTG	20	53.35	45.00	
EWB00_009712	GTAATGAACCTACTACTGTTGG	22	53.95	40.91	106
	GTTCGAGCCTCCTGATTG	18	54.90	55.56	
EWB00_006199	CAGTTGGAGAGCAAGGAG	18	54.90	55.56	107
	TGATGGACAGGAAGGAATAC	20	53.35	45.00	
EWB00_000274	GCAACTTCAAGAACCATACA	20	51.30	40.00	120
	ATTCCACTACGACCATCTC	19	53.01	47.37	
EWB00_006201	CCTGGTGAATCTGGACTTG	19	55.16	52.63	139
	CGCCTCTATCTCCTTCTAATC	21	55.61	47.62	

*Abbreviation*: Tm, melting temperature

### Statistical analysis

Data are presented as the mean ± standard deviation (SD). All statistical analyses were performed using a Student’s t-test. A probability (*P*) value of ≤ 0.05 was considered statistically significant. GraphPad Prism software (Version 6, GraphPad Software, La Jolla, CA, USA) was used for all statistical analyses.

## Results

### Worm burden, liver egg count and pathology

**Fig. 2:**
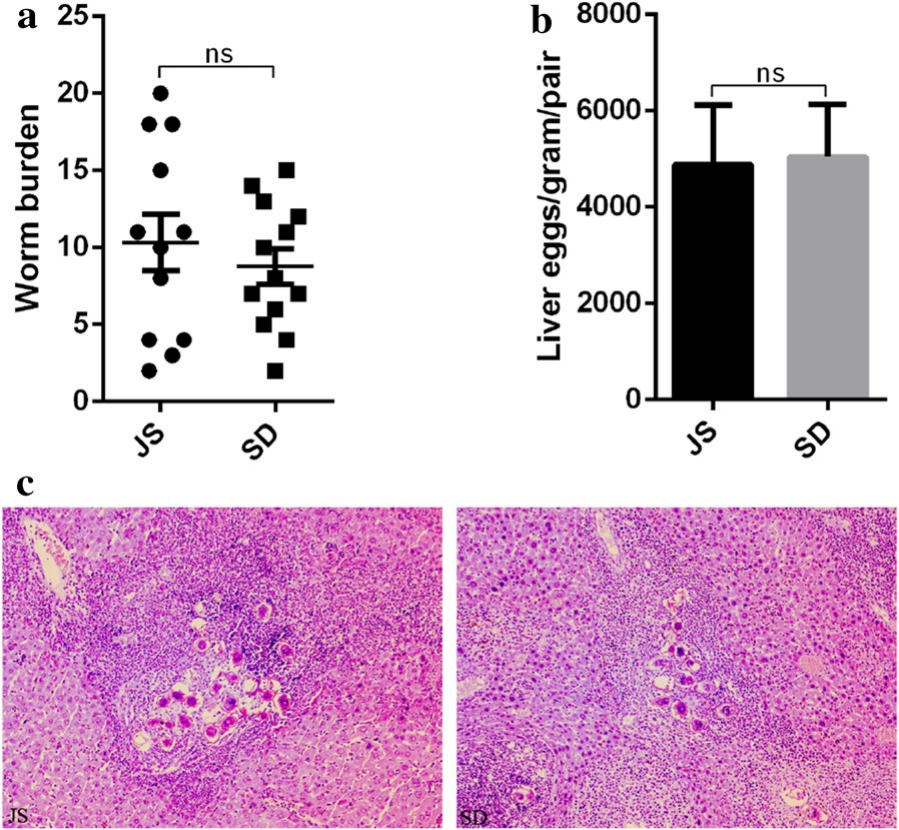
Pathogenicity to mice caused by *S. japonicum* derived from Jiangsu snails and Shandong snails. **a** Worm burden. **b** Liver egg count. **c** Liver pathology (original magnification: ×400). Data are expressed as the mean ± SD. Pairwise comparisons were performed with Student’s t-test (a *P*-value of ≤ 0.05 was considered statistically significant). *Abbreviations*: JS, Jiangsu snails; SD, Shandong snails; ns, not significant

Each mouse in the two groups was infected with 20 cercariae released from Jiangsu and Shandong snails, respectively. At 50 dpi, the adult worms and livers were collected from the two mice groups for comparing the worm burden, liver egg count and pathology. All mice in the two groups were successfully infected with schistosomes. The mean number of adult worms recovered from the two groups were 10.33 ± 1.83 (Jiangsu snails) and 8.77 ± 1.13 (Shandong snails) and the difference was not statistically significant (*t* = 0.74, *df* = 1, *P* = 0.47) (Fig. 2a). Among the selected mice, the mean number (± SD) of eggs per gram liver tissue per worm pair developed from cercariae released from Jiangsu and Shandong snails were 4886 ± 1231, 5046 ± 1088, respectively. Similarly, there was no significant difference (*t* = 0.10, df = 1, *P* = 0.93) (Fig. 2b).

H&E staining showed that there were large numbers of eosinophilic granulomatous nodules in the portal area caused by the eggs excreted from adult worms derived from Jiangsu and Shandong snails. The size of the nodules was different, and some of them had fibrosis contained clusters of *Schistosoma* eggs. A large quantity of eosinophils and small lymphocytes infiltrated around the eggs. The liver pathology in the two groups of mice was similar (Fig. 2c).

### Transcriptomic features of adult *S. japonicum* derived from *O. hupensis* bred in permissive and non-permissive areas

**Fig. 3:**
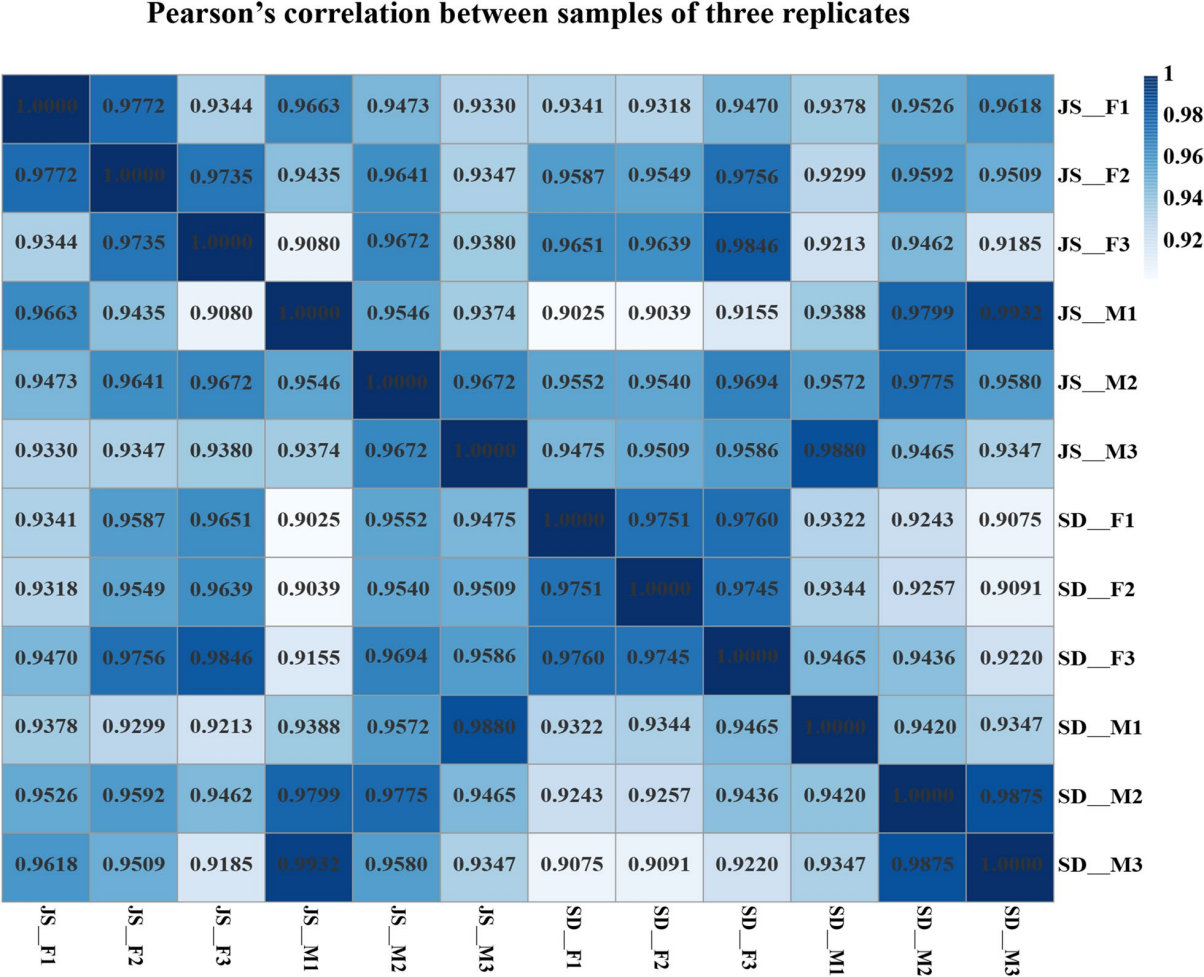
A heatmap showing Pearson’s correlation coefficient among different samples. The map indicates that all correlations between the two samples are positive and of moderate to large value

The RNA integrity number (RIN) of all twelve RNA samples used for RNA sequencing was > 7. A total of 575,398,346 raw reads were obtained from all twelve cDNA libraries. After eliminating adaptor and low-quality sequences, 570,783,338 clean reads were gained for the subsequent analysis. All clean reads were submitted to the Sequence Read Archive (SRA) database at NCBI (accession No. PRJNA579703). The Q30 percentage (Q30 percentage is the proportion of sequencing bases error rate with quality value < 0.1%) and GC percentage were 95.52% and 43.62%, respectively (Table 2). The alignment rate to the improved genome of *S. japonicum* ranged from 83.74% to 98.69%. Over 65% of the clean reads were distributed in exon regions and the rest were distributed in the introns. Pooled clean reads of all samples were mapped to a total of 8578 genes in the *S. japonicum* genome. Moreover, Pearson’s correlation coefficient of gene expression among different samples was greater than 0.9, indicating the high similarity of the gene expression patterns between samples (Fig. 3).

**Table 2:** Summary of transcriptome data of adult *S. japonicum* from different sources

Sample	Raw reads^a^	Raw bases^a^	Clean reads^a^	Clean bases^a^	Q30 (%)	GC content (%)
JS-F	141,998,818	21,441,821,518	140,909,612	21,080,375,081	95.26	42.68
JS-M	137,842,860	20,814,271,860	136,586,092	20,377,503,514	95.40	41.68
SD-F	153,538,068	23,184,248,268	152,271,370	22,807,757,670	95.88	48.74
SD-M	142,018,600	21,444,808,600	141,016,264	21,111,650,701	95.51	40.89
Total	575,398,346	86,885,150,246	570,783,338	85,377,286,966	95.52	43.62

^a^Reads of three replicates

### Analysis of differential expression

**Fig. 4:**
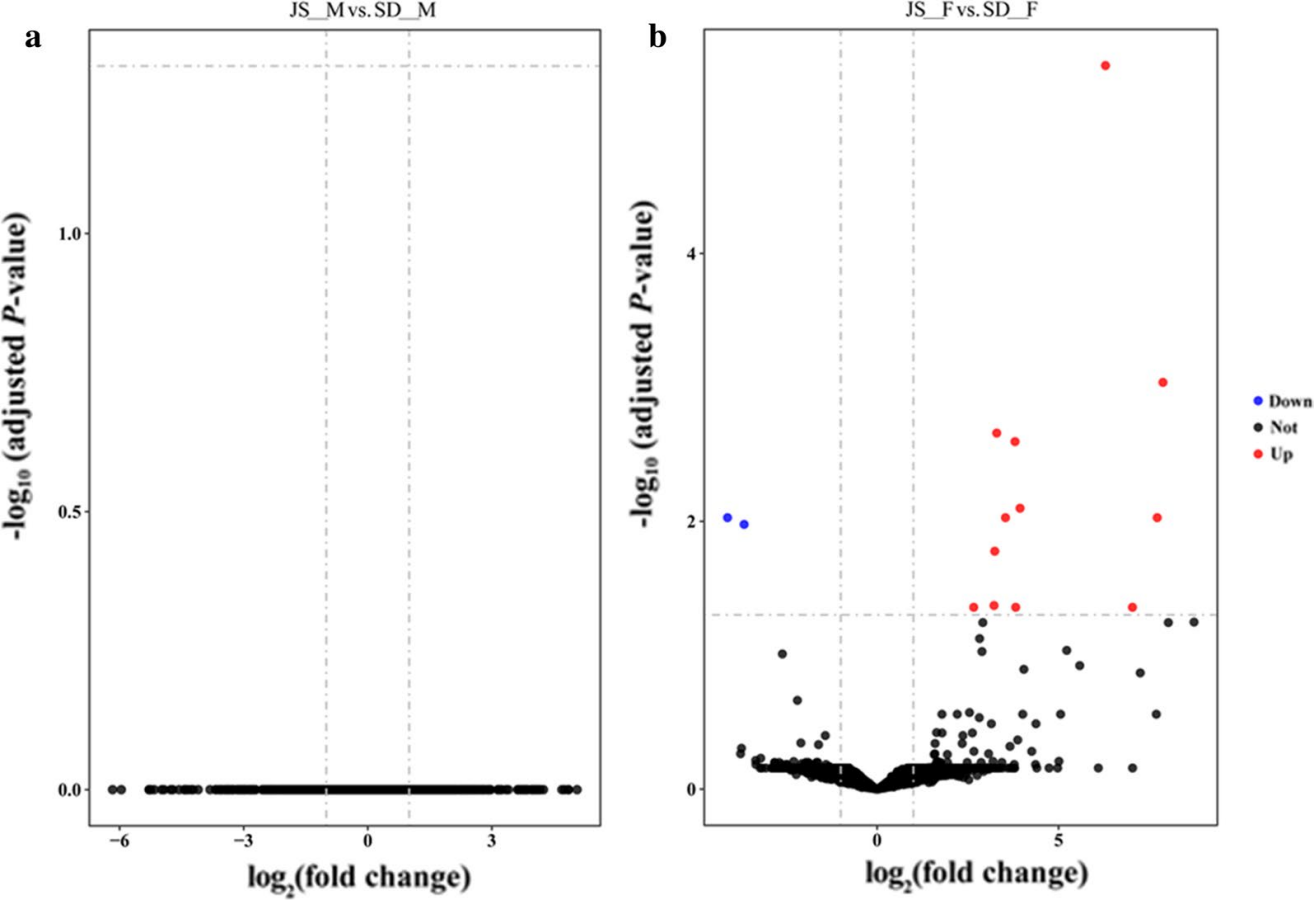
Volcano plots displaying the differentially expressed genes between adult *S. japonicum* derived from *O. hupensis* bred in permissive and non-permissive areas. **a** Differentially expressed genes between JS-M and SD-M. **b** Differentially expressed genes between JS-F and SD-F. Significantly differentially expressed genes are shown as red or blue (upregulated or downregulated in SD-F) dots. No significant difference between the expressions of genes is indicated by black dots. The X-axis and Y-axis represent the value of log_2_ (fold change) and the value of -log_10_ (adjusted *P*-value), respectively

In the present study, a threshold of FDR < 0.05 and absolute fold change ≥ 2 was used to define significantly differentially expressed genes in pairwise comparisons, including JS-M *vs* SD-M and JS-F *vs* SD-F. Of a total of 8578 genes, as shown in the volcano plots (Fig. 4), there was no differentially expressed gene between JS-M and SD-M. Similarly, only 14 genes were differentially expressed between JS-F and SD-F, of which 2 and 12 were significantly upregulated in JS-F and SD-F, respectively. The related information of the 14 differentially expressed genes is listed in Table 3. Interestingly, four of the 12 upregulated genes in SD-F are collagen-related genes (EWB00_010449, EWB00_006199, EWB00_000274 and EWB00_006201) and one of the downregulated genes is lactate dehydrogenase (EWB00_010161).

**Table 3:** Significantly differentially expressed transcripts in female worms derived from the two areas

Transcript ID	Description	Log2 (FC)	*P*-value	FDR	Upregulation
EWB00_005036	Hypothetical protein	− 4.12	1.15E-05	0.009	JS-F
EWB00_010161	l-lactate dehydrogenase A chain	− 3.66	1.46E-05	0.011	JS-F
EWB00_002418	SJCHGC02612 protein	7.87	2.82E-07	0.001	SD-F
EWB00_003584	Protein TAR1	7.72	1.16E-05	0.009	SD-F
EWB00_003582	10 kDa secreted	7.04	9.43E-05	0.044	SD-F
EWB00_008342	Small ubiquitin-related modifier 2	6.29	6.08E-10	3.95E-06	SD-F
EWB00_005617	Probable palmitoyltransferase ZDHHC11	3.94	6.14E-06	0.008	SD-F
EWB00_005592	Retrovirus-related Pol polyprotein from transposon 17.6	3.82	8.86E-05	0.044	SD-F
EWB00_010449	Collagen alpha-1(XXI) chain	3.80	1.56E-06	0.003	SD-F
EWB00_011245	Histone H2B	3.54	1.13E-05	0.009	SD-F
EWB00_009712	SJCHGC02190 protein	3.30	1.01E-06	0.002	SD-F
EWB00_006199	Collagen alpha-1(IV) chain	3.24	2.57E-05	0.017	SD-F
EWB00_000274	Collagen alpha-1(V) chain	3.22	7.19E-05	0.043	SD-F
EWB00_006201	Collagen-like protein	2.66	8.33E-05	0.044	SD-F

### qRT-PCR validation of RNA sequencing data

**Fig. 5:**
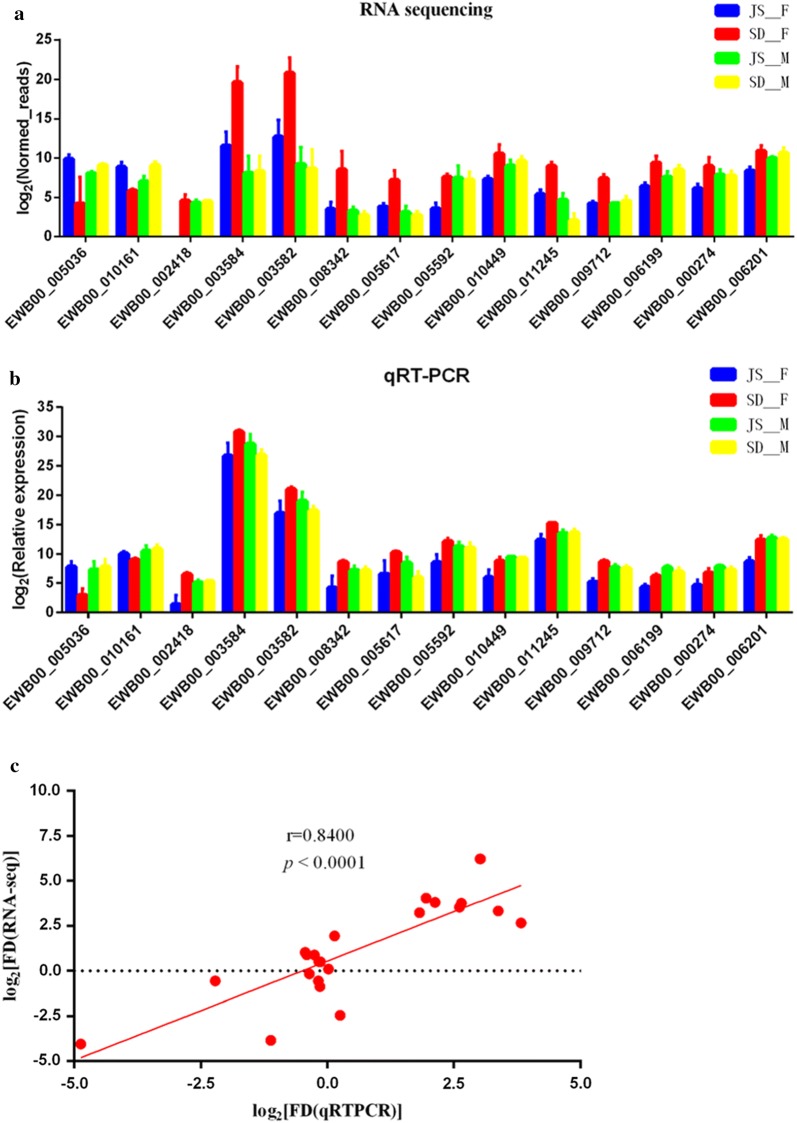
qRT-PCR validation of gene expression. Fourteen genes were selected for verification of the RNA sequencing data, including 2 genes upregulated in JS-F (EWB00_005036 and EWB00_010161) and 12 genes upregulated in SD-F (EWB00_002418, EWB00_003584, EWB00_003582, EWB00_008342, EWB00_005617, EWB00_005592, EWB00_010449, EWB00_011245, EWB00_009712, EWB00_006199, EWB00_000274 and EWB00_006201). PSMD4 was used for normalization. **a**, **b** Gene expression obtained from RNA sequencing and qRT-PCR, respectively. **c** The expression levels determined by qRT-PCR correlated with those obtained by RNA sequencing

To confirm the RNA sequencing data, 14 genes differentially expressed between JS-F and SD-F were further detected by qRT-PCR. According to the RNA sequencing results, the expression levels of EWB00_005036 and EWB00_010161 were upregulated in JS-F, the expression levels of EWB00_002418, EWB00_003584, EWB00_003582, EWB00_008342, EWB00_005617, EWB00_005592, EWB00_010449, EWB00_011245, EWB00_009712, EWB00_006199, EWB00_000274 and EWB00_006201 were upregulated in SD-F, and the expression levels of the 14 genes mentioned above were not significantly different between JS-M and SD-M. After normalizing to the reference gene PSMD4, expression levels determined by qRT-PCR correlated with those obtained by RNA sequencing (*r* = 0.8400, *P* < 0.0001), validating the accuracy and reliability of the RNA sequencing results (Fig. 5). Therefore, the data generated here can be used to investigate the difference in transcription levels between adult *S. japonicum* derived from *O. hupensis* bred in both Jiangsu and Shandong Province.

## Discussion

The SNWDP stretching across schistosomiasis endemic and non-endemic areas in China, may lead to the dispersal of *O. hupensis* from permissive areas to non-permissive areas in northern China. Our previous study showed *O. hupensis* can survive and breed for 13 years (12 generations) after being transferred to a non-permissive area, and maintain the susceptibility to *S. japonicum* infection (Miao et al., unpublished data) thus highlighting the extreme significance of investigation of the ability transferred snails to transmit *S. japonicum*.

In the present study, we first evaluated the difference in pathogenicity to mice, using cercariae of *S. japonicum* derived from the transferred *O. hupensis* bred in non-permissive areas (Jining City of Shandong Province) and cercariae released from the snails from the original habitat (Jiangsu Province). The results indicated no significant differences between the two groups regarding worm burden, liver egg count and pathology. Furthermore, to explore the differences at the molecular level of the worms developed from cercariae released from Jiangsu and Shandong snails, RNA sequencing technology was used for exploring gene expression profiles of adult *S. japonicum* (at 50 dpi) derived from the two snail groups. Analysis of differential gene expression showed that no gene was differentially expressed between adult male groups (JS-M *vs* SD-M) and only 14 genes were differentially expressed between adult female groups (JS-F *vs* SD-F). This result is not surprising. He et al. [36] found that there was no significant genetic divergence between the snail population in Lake Weishan and its original population in River Yangtze using microsatellite DNA markers, 10 years after migration. These results reconfirm the lack of change in pathogenicity and slight changes in the transcriptional levels of the adult worms in the present study. On the other hand, the differentially expressed genes in males derived from snails bred in permissive and non-permissive areas were fewer than in females, which may be related to the fact that the gene expression in males is more stable than that in females in the course of development [37].

Of the 14 differentially expressed genes, four of the 12 highly expressed genes in SD-F are collagen-related genes (EWB00_010449, EWB00_006199, EWB00_000274, EWB00_006201). Collagen proteins either become cross-linked into high-strength mature fibers and are distributed in the spaces around the cells or form heterotypic fibrils, distributing in the bones, muscles, tendons, ligaments, skin, cornea and other parts of the organism [38, 39]. Besides their structural roles, collagens also have numerous potential functions during growth, repair and morphogenesis, and in pathobiological processes. RNAi silencing of type V collagen in *S. japonicum* was shown to significantly reduce the hatching rate of eggs and single female spawning rate and also affect the morphology of the worms [40]. One of lowly expressed genes (EWB00_010161) in SD-F is lactate dehydrogenase (LDH), encoding a terminal glycolytic enzyme that catalyzes the interconversion of pyruvate and lactate in the presence of the nicotinamide adenine dinucleotide coenzyme. Schistosome parasitic stages depend mainly on anaerobic energy metabolism and glycolytic enzymes are essential for the survival of parasites [41, 42]. Four genes (EWB00_005036, EWB00_003584, EWB00_003582 and EWB00_009712) have no functional domains or unknown domains. The remaining of the highly expressed genes in SD-F are involved in protein modification and transcriptional regulation. In the process of transcriptome sequencing, the changes of the 14 genes were negligible. The materials used for RNA sequencing were from the same group of mice at the same time and the only different factor was the snail origin. Whether the differentially expressed genes are related to the intermediate host, *O. hupensis*, remains to be further investigated.

Additionally, we performed qRT-PCR to detect the 14 differentially expressed genes for validating the RNA sequencing data and found that the consistency between the two methods was acceptable. To obtain better measurements, there may be a need to further increase the sequencing depth and include more biological replicates for providing greater statistical power.

This study represents a snapshot of comparative transcriptome analysis for adult *S. japonicum* (at 50 dpi) derived from *O. hupensis* bred in Jiangsu Province and Shandong Province. In future studies, we could collect sporocysts, freshly released cercariae or schistosomula to carry out a comparative analysis of gene expression. It is possible that more differences at the molecular level among the comparison groups will be found. In addition, the same effect may be achieved by adding different perspectives of investigation, such as epigenetic analysis or protein modification. On the other hand, considerable variation between some of *S. japonicum* populations has been reported for genes inferred to be linked to key cellular processes and/or pathogen-host interactions [43]. In our study, we only used one population in the infection experiment which showed no significant differences. However, it is possible to find some differences if using different populations of worms.

*Oncomelania hupensis* transferred from Jiangdu County (Jiangsu Province) to Lake Weishan (Shandong Province) could survive and spawn for 13 years (12 generations) after migration. The offspring of transferred snails still maintained the susceptibility to *S. japonicum* infection. In this study, we provide further evidence that the pathogenicity of cercariae released by the snails remained unchanged. Moreover, the adult *S. japonicum* derived from *O. hupensis* transferred from permissive to non-permissive areas changed only slightly at the transcriptomic level. Altogether, these results suggest that a long-term surveillance system of schistosomiasis should be established for large-scale water conservancy projects, especially those which pass through schistosomiasis endemic areas and non-endemic areas, to prevent the diffusion of snails and reduce the risk of schistosomiasis transmission.

## Conclusions

No difference in pathogenicity to mice was observed between the cercariae of *S. japonicum* from *O. hupensis* bred in Jiangsu and Shandong Province, two sites located on the ERP of SNWDP that represent permissive and non-permissive areas for snail breeding, respectively. Unsurprisingly, transcriptome profiles of adult *S. japonicum* male and female worms (at 50 dpi) derived from the two groups of snails revealed that only 0 and 14 significantly differentially expressed genes were obtained between the comparison groups of male and female worms, respectively. Interestingly, four of the 14 differentially expressed genes between female comparison groups were collagen-related genes and one was lactate dehydrogenase (LDH). However, whether these differentially expressed genes are associated with the intermediate snail hosts, remains to be further investigated. Combined with the results of our previous study, we suggest that *O. hupensis* transferred from permissive to non-permissive areas retain the original transmission capacity of schistosomiasis. Therefore, a long-term surveillance system of schistosomiasis along the ERP of SNWDP should be constructed to prevent the spread of snails and reduce the risk of schistosomiasis transmission.

## Data Availability

The data supporting our findings and conclusions are included within the article. The clean data of RNA sequencing are available at NCBI (accession No.: PRJNA579703).

## Abbreviations

ERP: East Route Project
SNWDP: South-to-North Water Diversion Project
dpi: days post-infection
qRT-PCR: quantitative real-time PCR
LDH: lactate dehydrogenase
JS-M: adult male worms derived from infected Jiangsu snails
JS-F: adult female worms derived from infected Jiangsu snails
SD-M: adult male worms derived from infected Shandong snails
SD-F: adult female worms derived from infected Shandong snails
FDR: false discovery rate
RIN: RNA integrity number
PSMD4: 26S proteasome non-ATPase regulatory subunit 4

## References

[1] Colley DG, Bustinduy AL, Secor WE, King CH (2014). Human schistosomiasis. Lancet..

[2] Barsoum RS, Esmat G, El-Baz T (2013). Human schistosomiasis: clinical perspective: review. J Adv Res..

[3] Yang GJ, Liu L, Zhu HR, Griffiths SM, Tanner M, Bergquist R (2014). Chinaʼs sustained drive to eliminate neglected tropical diseases. Lancet Infect Dis..

[4] Yang Y, Zhou YB, Song XX, Li SZ, Zhong B, Wang TP (2016). Integrated control strategy of schistosomiasis in The Peopleʼs Republic of China: projects involving agriculture, water conservancy, forestry, sanitation and environmental modification. Adv Parasitol..

[5] Zhang LJ, Xu ZM, Dai SM, Dang H, Lv S, Xu J (2018). Endemic status of schistosomiasis in People’s Republic of China in 2017. Zhongguo Xue Xi Chong Bing Fang Zhi Za Zhi..

[6] Zhao QP, Jiang MS, Littlewood DT, Nie P (2010). Distinct genetic diversity of *Oncomelania hupensis*, intermediate host of *Schistosoma japonicum* in mainland China as revealed by ITS sequences. PLoS Negl Trop Dis..

[7] Mao SB (1990). Biology of schistosome and control of schistosomiasis.

[8] Wang L, Utzinger J, Zhou XN (2008). Schistosomiasis control: experiences and lessons from China. Lancet..

[9] Yuan Y, Xu XJ, Dong HF, Jiang MS, Zhu HG (2005). Transmission control of schistosomiasis japonica: implementation and evaluation of different snail control interventions. Acta Trop..

[10] Zhou XN, Bergquist R, Leonardo L, Yang GJ, Yang K, Sudomo M (2010). Schistosomiasis japonica control and research needs. Adv Parasitol..

[11] Seto EY, Wu W, Liu HY, Chen HG, Hubbard A, Holt A (2008). Impact of changing water levels and weather on *Oncomelania hupensis hupensis* populations, the snail host of *Schistosoma japonicum*, downstream of the Three Gorges Dam. EcoHealth..

[12] Hu Y, Li R, Bergquist R, Lynn H, Gao FH, Wang QZ (2015). Spatio-temporal transmission and environmental determinants of schistosomiasis japonica in Anhui Province. China. PLoS Negl Trop Dis..

[13] WHO (1985). The control of schistosomiasis.

[14] Talla I, Kongs A, Verle P, Belot J, Sarr S, Coll AM (1990). Outbreak of intestinal schistosomiasis in the Senegal River Basin. Ann Soc Belg Med Trop..

[15] Audibert M, Josseran R, Josse R, Adjidji A (1990). Irrigation, schistosomiasis, and malaria in the Logone Valley. Cameroon. Am J Trop Med Hyg..

[16] Hunter JM, Rey L, Scott D (1982). Man-made lakes and man-made diseases. Towards a policy resolution. Soc Sci Med..

[17] South-North Water Transfer Project. Wikipedia. 2019. https://en.wikipedia.org/wiki/South%E2%80%93North_Water_Transfer_Project. Accessed 23 Feb 2019.

[18] Liang YS, Wang W, Li HJ, Shen XH, Xu YL, Dai JR (2012). The South-to-North Water Diversion Project: effect of the water diversion pattern on transmission of *Oncomelania hupensis*, the intermediate host of *Schistosoma japonicum* in China. Parasit Vectors..

[19] Zhou XN (2005). Science on *Oncomelania* snail.

[20] Wang W, Dai JR, Liang YS, Huang YX, Coles GC (2009). Impact of the South-to-North Water Diversion Project on the transmission of *Schistosoma japonicum* in China. Ann Trop Med Parasitol..

[21] Xu J, Li SZ, Huang YX, Cao ZG, Tu ZW, Wu CG (2012). Risk evaluation of schistosomiasis japonica in potential endemic areas in China. Zhongguo Ji Sheng Chong Xue Yu Ji Sheng Chong Bing Za Zhi..

[22] Zhu YF, Gao JB, Huang YM, Kuang RX, Hang DR, He Y (2013). Surveillance of endemic situation of schistosomiasis in Gaoyou sections in east route of South-to-North Water Diversion Project before water transfer. Zhongguo Xue Xi Chong Bing Fang Zhi Za Zhi..

[23] Huang YX, Li TC, Hang DR, Sun DK, Zheng B, Zhang JF (2012). Study on potential risks of schistosomiasis transmission in Grand Canal West water diversion route of eastern route project of South-to-North Water Diversion Project. Zhongguo Xue Xi Chong Bing Fang Zhi Za Zhi..

[24] Sun DK, Li Q, Wang QL (2011). Surveillance of schistosomiasis in Jinbao Channel areas in east route of South-to-North Water Diversion Project. Zhongguo Xue Xi Chong Bing Fang Zhi Za Zhi..

[25] Li W, Gao JB, Zheng B, Huang YX, Hang DR, Zhang JF (2011). Surveillance of *Oncomelania hupensis* snails in Baoying and Gaoyou sections of Li Canal in east route of South-to-North Water Diversion Project. Zhongguo Xue Xi Chong Bing Fang Zhi Za Zhi..

[26] Miao F, Wang YB, Bu XQ, Liu X (2017). Longitudinal observation on the reproduction and survivability of the north-migrating *Oncomelania hupensis* in Dongping Lake, Shandong Province. J of Pub Health and Prev Med..

[27] Wang W, Liang YS, Dai JR, Huang YX (2008). Impact of the construction of the South-to-North Water Diversion Project on distribution of *Oncomelania hupensis*, the intermediate host of *Schistosoma japonicum* in China. Acta Ecol Sinica..

[28] Miao F, Li WQ, Liu YC, Wen PE (2003). Study on the viability of *Oncomelania hupensis* on irrigation network in Shandong Province after reversion of Yangtze River to arid northern part of China. China Trop Med..

[29] Bolger AM, Lohse M, Usadel B (2014). Trimmomatic: a flexible trimmer for Illumina sequence data. Bioinformatics..

[30] Luo F, Yin MB, Mo XJ, Sun CS, Wu QF, Zhu BK (2019). An improved genome assembly of the fluke *Schistosoma japonicum*. PLoS Negl Trop Dis..

[31] Kim D, Langmead B, Salzberg SL (2015). HISAT: a fast spliced aligner with low memory requirements. Nat Methods..

[32] Anders S, Pyl PT, Huber W (2015). HTSeq - a Python framework to work with high-throughput sequencing data. Bioinformatics..

[33] Anders S, Huber W (2010). Differential expression analysis for sequence count data. Genome Biol..

[34] Liu S, Cai PF, Hou N, Piao XY, Wang H, Hung T (2012). Genome-wide identification and characterization of a panel of house-keeping genes in *Schistosoma japonicum*. Mol Biochem Parasitol..

[35] Livak KJ, Schmittgen TD (2001). Analysis of relative gene expression data using real-time quantitative PCR and the 2(-Delta Delta C(T)) method. Methods..

[36] He J, Miao F, Yang K, Zhao CL, Liu X (2016). Genetic variation of *Oncomelania hupensis* in Weishan Lake of Shandong Province using microsatellite DNA markers. Zhongguo Ji Sheng Chong Xue Yu Ji Sheng Chong Bing Za Zhi..

[37] Wang JP, Yu Y, Shen HM, Qing T, Zheng YT, Li Q (2017). Dynamic transcriptomes identify biogenic amines and insect-like hormonal regulation for mediating reproduction in *Schistosoma japonicum*. Nat Commun..

[38] Birk DE (2001). Type V collagen: heterotypic type I/V collagen interactions in the regulation of fibril assembly. Micron..

[39] van der Rest M, Garrone R (1991). Collagen family of proteins. FASEB J..

[40] Yang YX, Jin YM, Liu PP, Shi YL, Cao YF, Liu JM (2012). RNAi silencing of type V collagen in *Schistosoma japonicum* affects parasite morphology, spawning, and hatching. Parasitol Res..

[41] Lu G, Hu XC, Peng ZY, Xie HY, Li YW, Wu ZD (2006). Expression and characterization of lactate dehydrogenase from *Schistosoma japonicum*. Parasitol Res..

[42] Guerra-Sa R, Franco GR, Pena SD, Rodrigues V (1998). Lactate dehydrogenase: sequence and analysis of its expression during the life cycle of *Schistosoma mansoni*. Mem Inst Oswaldo Cruz..

[43] Young ND, Chan K, Korhonen PK, Min Chong T, Ee R, Mohandas N (2015). Exploring molecular variation in *Schistosoma japonicum* in China. Sci Rep..

